# Targeting allosteric sites of human aromatase: a comprehensive *in-silico* and *in-vitro* workflow to find potential plant-based anti-breast cancer therapeutics

**DOI:** 10.1080/14756366.2021.1937145

**Published:** 2021-06-17

**Authors:** Hani A. Alhadrami, Ahmed M. Sayed, Sami A. Melebari, Asem A. Khogeer, Wesam H. Abdulaal, Mohamed B. Al-Fageeh, Mohammad Algahtani, Mostafa E. Rateb

**Affiliations:** a Department of Medical Laboratory Technology, Faculty of Applied Medical Sciences, King Abdulaziz University, Jeddah, Saudi Arabia; b Molecular Diagnostic Lab, King Abdulaziz University Hospital, King Abdulaziz University, Jeddah, Saudi Arabia; c Molecular Diagnostic Unit, The Regional Laboratory in Makkah, Ministry of Health, Makkah, Kingdom of Saudi Arabia; d Department of Pharmacognosy, Faculty of Pharmacy, Nahda University, Beni Suef, Egypt; e Plan and Research Department, General Directorate of Health Affairs, Makkah region, Ministry of Health, Makkah, Kingdom of Saudi Arabia; f Department of Biochemistry, Faculty of Science, King Abdulaziz University, Jeddah, Saudi Arabia; g General Directorate for Funds and Grants (GDFG), King Abdulaziz City for Science and Technology, Riyadh, Saudi Arabia; h Department of Laboratory and Blood Bank, Security Forces Hospital Program, Mecca, Saudi Arabia; i School of Computing, Engineering & Physical Sciences, University of the West of Scotland, Paisley, UK

**Keywords:** Aromatase, anti-breast cancer, allosteric inhibition, natural products, *in silico*

## Abstract

Recent findings suggested several allosteric pockets on human aromatase that could be utilised for the development of new modulators able to inhibit this enzyme in a new mechanism. Herein, we applied an integrated *in-silico*-based approach supported by *in-vitro* enzyme-based and cell-based validation assays to select the best leads able to target these allosteric binding sites from a small library of plant-derived natural products. Chrysin, apigenin, and resveratrol were found to be the best inhibitors targeting the enzyme’s substrate access channel and were able to produce a competitive inhibition with IC_50_ values ranged from 1.7 to 15.8 µM. Moreover, they showed a more potent antiproliferative effect against ER+ (MCF-7) than ER- one (MDA-MB-231) cell lines. On the other hand, both pomiferin and berberine were the best hits for the enzyme’s haem-proximal cavity producing a non-competitive inhibition (IC_50_ 15.1 and 21.4 µM, respectively) and showed selective antiproliferative activity towards MCF-7 cell lines.

## Introduction

Breast cancer (BC) is considered the top reported malignancy among women and currently ranks the second after lung cancer in female cancer-related deaths^1^. It is obvious now that BC has different characterising biological subtypes (i.e. heterogeneous disease), and hence, other therapeutic choices can be provided depending on the tumour genetic profile^2^. Oestrogen receptor-positive (ER+) BC is the most prevailing subtype (∼75% of the reported cases) that requires oestrogen for its development and progression. Thus, Selective Oestrogen Receptor Modulators (SERM), like tamoxifen, have been introduced as a therapeutic agent to control such type of BC and to prevent their relapse^3^. Currently, drugs that block the aromatisation of androgens to oestrogen via inhibition of a key synthesising enzyme namely cytochrome P450 (CYP450) aromatase (human aromatase; HA) are considered the first-line choice for the management of ER + BC (e.g. letrozole and anastrozole; 1 and 2)^4^. Despite the efficacy of SERM and aromatase inhibitors (ArIs) as treatment options, complete deprivation of oestrogen that induced by such agents is associated with several side effects (e.g. osteoporosis and menopausal symptoms). Additionally, such abrupt loss of oestrogen may also accelerate the development of resistance leading to rapid disease relapse^5^. Reporting of the first crystallised HA in 2009 has opened the door for extensive investigation to find new HA inhibitors^6^ that mainly act as competitive ones^7–9^. Recently, Ghosh and co-workers have reported a new crystallised HA indicating the presence of an allosteric binding site (i.e. haem-proximal cavity) on HA that might act as a regulator for its activity by interacting with NADPH cytochrome P450 reductase (CPR) that provide the HA’s haem moiety with the electrons necessary for the reduction process^10^. Later on, Spinello et al., utilized this newly characterised allosteric site to discover new non-competetive HA inhibitors that were able to down-regulate the enzyme’s activity without complete blockage of oestrogen production. Hence, such balanced inhibition of HA might reduce the side effects caused by common ArIs and delay the onset of resistance^11^. Moreover, the substrate access channel was also found to be a crucial binding site for HA’s competitive inhibitors and should be utilised in structure-based drug design rather than the sequestered enzyme’s active site^11^^,^^12^.

Natural products still represent a potential pipeline of new leads in drug discovery. Several plant-derived natural products have shown potent activity against HA, notably flavonoids that were reported to exhibited competitive inhibition against HA^13–15^. However, the exact mode of inhibition of such class of natural products remained elusive. In addition, many natural products-based lead compounds failed to be promising drug candidates due to their poor drug-like and pharmacokinetic properties^16^.

In this context, we proposed an integrated in-silico and in-vitro pipeline to find out potential HA inhibitors from a plant-based natural products library proposing their exact binding site and modes of inhibition.

The compounds library prepared for this study included 52 different plant metabolites from several major classes of plant metabolites (e.g. flavonoids, phenolics, stilbenes, alkaloids, terpenes, and sterols). Phenolics and flavonoids were the most represented class (63.5%) as they has been reported previously to be HA-specific inhibitors^13–15^ and they are also considered the most abundant metabolites among other plant natural products.

All of these metabolites are considered major metabolites in their corresponding plant source so that they can be readily purified for further processing. Hence, this library is considered a good representative for the common plant natural products. Additionally, it shows a good structural diversity to test the efficacy of our proposed in silico protocol in differentiating between active and inactive compounds.

**Figure 1.**
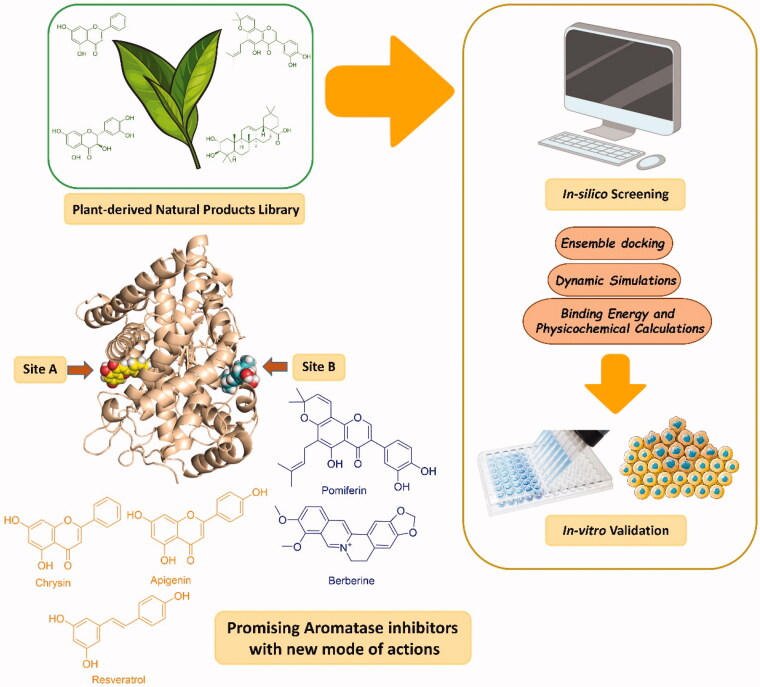
The approach applied in the present investigation.

We utilised this library in a protocol that integrates a number of computational and experimental steps as the following: (i) structure-based virtual screening (VS) using our in house library of plant-derived natural products (52 compounds); (ii) selecting top-scoring hits that obey Lipinski’s rule of five^16^; (iii) 100 ns molecular dynamic simulations (MDS) to refine docking poses of the top-scoring compounds indicating the binding free energies of the most stable hits; (iv) validation of the computational studies by enzyme and cell-based *in vitro* assays. Our findings confirmed that five compounds were found to inhibit HA in the low µM range through competitive and non-competitive mechanisms that putatively established by targeting allosteric sites (Sites A and B) on the enzyme. Consequently, our outcomes in the present study highlighted the potential of natural products-based therapeutics for ER + BC that probably able to delay or avoid the onset of resistance which frequently develops with the current therapies. The approach applied in this study is depicted in Figure 1.

## Materials and methods

### Library construction

All compounds used in this study (Figure S1) were purchased (compounds: 1–11, 13, 15, 17, 18–22, 26–29, 31–33, 36–40, 42–47, Alfa Aesar, Massachusetts, USA and Sigma-Aldrich, Saint Louis, USA) or isolated from their natural source following the previously reported procedures^17–29^. The constructed plant-based small library of the present study consisted of 18 phenolic compounds (1–8, 11–19, 24), 10 non-glycosylated flavonoids (i.e. aglycones; 27–36), 5 glycosylated flavonoids (37–41), 5 alkaloids (9, 10, 25, 26, 52), 5 steroids (45–49), 5 terpenoids (42–44, 50, 51), and 4 quinone-derived compounds (20–23).

### In silico screening

#### Ensemble docking

Docking experiments were performed using AutoDock Vina software^30^. All the prepared library’s compounds were docked against both the substrate access channel (Site A) and haem-proximal cavity (Site B) of HA (PDB code: 5JKV) that its crystal structure was downloaded from protein databank (https://www.rcsb.org/). A list of the residues of these binding sites together with their grid boxes is reported in Table 1S. To account for these binding sites’ flexibility, we used their MDS-derived conformers sampled every 10 ns for docking experiments (i.e. ensemble docking). Subsequently, the retrieved top hits were ranked according to their binding energies. The generated docking poses were visualised and analysed using Pymol software^30^.

**Table 1.** Inhibitory activities (IC_50_ and *K*_i_) of the top- and low-scoring compounds against HA, MCF-7, and MDA-MB-231 indicating their binding free energies and mode of HA inhibition.

Compound	HA (*K_i_*)*	MCF-7	MDA-MB-231	ΔG**	Binding site	Mode of inhibition
Chrysin	1.7 ± 1.4 (1.1 ± 1.2)	7.8 ± 2.8	65 ± 2.3	−9.9	Site-A	Competitive inhibitor
Apigenin	4.8 ± 1.1 (3.5 ± 0.6)	13.6 ± 3.5	60 ± 1.6	−9.8	Site-A	Competitive inhibitor
Resveratrol	15.8 ± 0.9(10.3 ± 1.4)	11 ± 2.2	38 ± 3.5	−9.5	Site-A	Competitive inhibitor
Tangeretin	> 100	30.3 ± 2.1	45.6 ± 3.8	−5.9	Site-A	–
Tanshinone IIA	42.5 ± 1.2 (40.9 ± 1.4)	1.4 ± 1.3	3.7 ± 1.7	−6.1	Site-B	Non-competitive
Pomiferin	15.1 ± 0.5 (14.8 ± 0.6)	5.4 ± 2.2	30.9 ± 3.1	−12.2	Site-B	Non-competitive
Betulin	95.3 ± 1.1 (93.8.5 ± 0.9)	67 ± 2.6	>100	−6.2	Site-B	Non-competitive
Berberine	21.4 ± 0.9 (20.1 ± 0.4)	10.2 ± 2.6	>100	−10.9		Non-competitive
Abscisic acid^#^	>100	>100	>100	> −3	–	–
Rutin^#^	>100	>100	>100	> −3	–	–
Letrozole^##^	0.03 ± 0.01 (0.02 ± 0.01)	5.6 ± 1.1	41.3 ± 2.1	–	–	Competitive inhibitor

*Values inside parenthesis are of the inhibition constant (*K*_i_).

**Binding free energies (ΔG) calculated during MDS.

^#^Representing compounds that got low ΔG to test the accuracy of our *in silico* protocol.

^##^Reference HA inhibitor.

### Molecular dynamic simulation

MD simulations were performed by Desmond v. 2.^2^^,^^31^^,^^32^ the MDS machine of Maestro software^33^ using the OPLS3 force field. HA (PDB code: 5JKV) systems were built via System Builder option, where it was embedded in an orthorhombic box of TIP3P waters together with 0.15 M Na^+^ and Cl^-^ ions with 20 A˚ solvent buffer from the molecular surface of the centrally placed receptor. Afterwards, the prepared system was energy minimised and equilibrated for 10 ns. Desmond software automatically parameterises inputted ligands during the system building step according OPLS force field. For simulations performed by NAMD, the parameters and topologies of the compounds were calculated either using Charmm27 force field by the online software Ligand Reader & Modeller (http://www.charmm-gui.org/?doc=input/ligandrm)^34^ or by the VMD plugin Force Field Toolkit (ffTK)^35^. Afterward, the generated parameters and topology files were loaded to VMD so that it can readily read the protein-ligand complexes without errors and then conduct the simulation step.

Simulations were run for 100 ns at 310 K in the NPT ensemble with the Nose-Hoover thermostat and Martyna-Tobias-Klein barostat using an anisotropic coupling. We used the best binding poses for each compound inside both Site A and Site B as starting systems to investigate their binding stability and mode of interactions.

Binding free energy calculations (Δ*G*) were performed using the free energy perturbation (FEP) method. We first prepared the input files and script NAMD by the online-based software CHARMM-GUI Free Energy Calculator (http://www.charmm-gui.org/input/fec). Afterwards, these inputs were loaded to NAMD for simulations, where the equilibration was performed in the NPT ensemble at 310 K and 1 atm (1.01325 bar) with Langevin piston pressure (for ″Complex″ and ″Ligand″) in the presence of TIP3P water model. 10 ns FEP simulations were performed for each system, and the frames of the last 5 ns of the free enegry were measured for the final free energy values^36^. For protein-ligand complexes, we used their docking poses as starting structures.

Retrieved Δ*G* values were further cross-validated using Desmond software by also applying FEP method using OPLS3 force field according the default protocol: the systems were solvated in SPC water molecules with widths of 10 Å for the complexes and 15 Å for the solvent simulations. Afterwards, the prepared systems allowed to be relaxed and equilibrated using the default protocol od Desmond, consisting of an energy minimisation and then 12 ps length simulations at 10 K using an NVT ensemble, followed by an NPT ensemble. Subsequently, the restrained system was equilibrated at room temperature using the NPT ensemble. Finally, a 240 ps room-temperature NPT ensemble simulation was performed. 5 ns production simulations in the NPT ensemble were performed for both the complex and the solvent systems^31^^,^^32^.

For further confirmation of the initial docking and MDS experiments, we generated a binding event simulation by placing the 5 ligands close to each binding site (∼15 Å) and applying force (10 kcal/mol.A^2^) towards the haem moiety to make each ligand moving towards each binding site with a velocity 0.75 Å/ns. Finally, generated trajectories were visualised and analysed by VMD software^35^.

### ADME properties and shape complementarity scores calculations

Absorption, Distribution, Metabolism, and Excretion (ADME) properties was calculated using the online website “http://www.swissadme.ch/”^37^. Gastrointestinal (GIT) absorption, blood–brain barrier (BBB), solubility, bioavailability score, and inhibition of CYP2D6 were selected as ADME descriptors to be calculated. In addition, drug-likeness of each compound was suggested depending on their adherence to Lipinski’ rules^16^. In regard to shape complementarity scores calculations, we used the algorithm of Lawrence and Colman, which calculate the degree of geometric fit between the surfaces of two entities^38^.

### *In-vitro* validation

#### Enzyme inhibition assay

HA inhibition assay was performed using the Aromatase Inhibitor Screening Kit (BioVision Inc., San Francisco, USA), which utilised a fluorogenic substrate that is converted by HA into a highly fluorescent metabolite. After reconstitution of the kit’s reagents, a standard curve was constructed a serial dilution of the fluorescent standard. Each test compound was dissolved in DMSO to reach a final concentration of 0.25% (*v/v*). Subsequently, each solution was diluted in the aromatase assay buffer to obtain a range of concentrations for constructing a dose-response curve. The reaction was conducted by mixing aromatase mix (containing Recombinant Human Aromatase (2X), Aromatase assay buffer and NADPH-generating system) with the test compound. The reaction mixture was pre-incubated for 10 min at 37 °C to allow the test compounds to bind to HA. Then, the reaction was initiated after adding 30 ml of aromatase substrate/NADP^+^ mixture (containing buffer, aromatase substrate and ß-NADPþ 100X stock). The assays were conducted in 96-well microtiter plates in a final reaction volume of 100 μL/well. The resulted fluorescence was measured using a microplate reader (BioTek Synergy, Germany; dual wavelengths of 485/535) for 60 min. Experiments were performed in triplicate, and the average values were used to generate the dose response curves. To calculate the concentration that was able to inhibit HA activity by 50% (IC_50_), each compound was tested at different concentrations (200, 100, 50, 10, 1 and 0.1 µM). In order to analyse the inhibition mechanism of each inhibitor, we performed a number of kinetic experiments at constant test compound (set at 30% of their IC_50_ values) and at different substrate concentrations ranging from 0.25 to 6 times the approximate value of Km. For competitive inhibition, the % inhibition decreases as the substrate concentration increases, while it remains constant for the non-competitive one^38^. The inhibition constant (*K*_i_) values for each inhibitor were determined according to the manufacturer protocol, where the rate of substrate utilisation, using 2 mM of the tested enzyme and 0–250 µM of the substrate, was monitored in increasing amounts of inhibitor (0–50 µM).

#### Antiproliferative assay

The cell lines MCF-7 and MDA-MB- 231 used in this investigation were obtained from the American Type Culture Collection (ATCC, Rockville, MD, USA). MCF-7 cell proliferation was induced by testosterone. Tumour cells were seeded in triplicate in 12-well plates. 24 h later, they exposed for 72 h to an increasing concentration of each tested compound (from 1 to 200 µM) together with 10 µM testosterone undecanoate. Tumour cells were harvested using trypsin and counted. Three independent experiments were performed, and the concentration of the compound able to inhibit cell growth by 50% (IC_50_) was calculated.

## Results

### Substrate access channel-directed inhibition (Site A)

**Figure 2.**
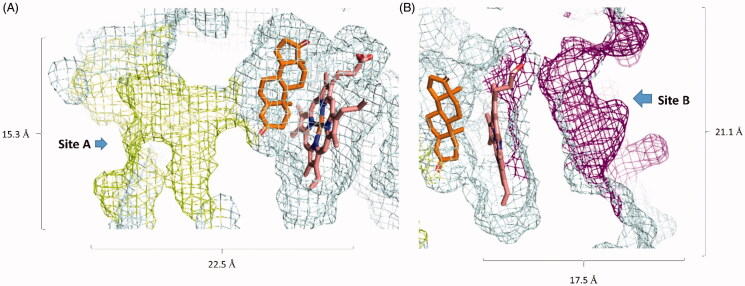
Geometry and dimensions of both Site A and Site B. A: The substrate access channel, where the lemon yellow colour indicates Site A, the orange compound is an HA’s substrate (androstenedione), and the brick-red compound is the haem moiety. B: is an HA’s allosteric site, where the purple colour is Site B.

HA’s substrate access channel is a 22.5 Å long channel with a narrow opening (i.e. Site A) and extends to reach a haem moiety at the end, where the synthesis of oestrogen occurs. It has a total volume of 2351 Å^3^, and normally the substrate needs to cross this channel to reach to the haem moiety in order to be catalysed (Figure 2)^10^. Hence, targeting this site will lead eventually to a competitive-type of HA inhibition.

**Figure 3.**
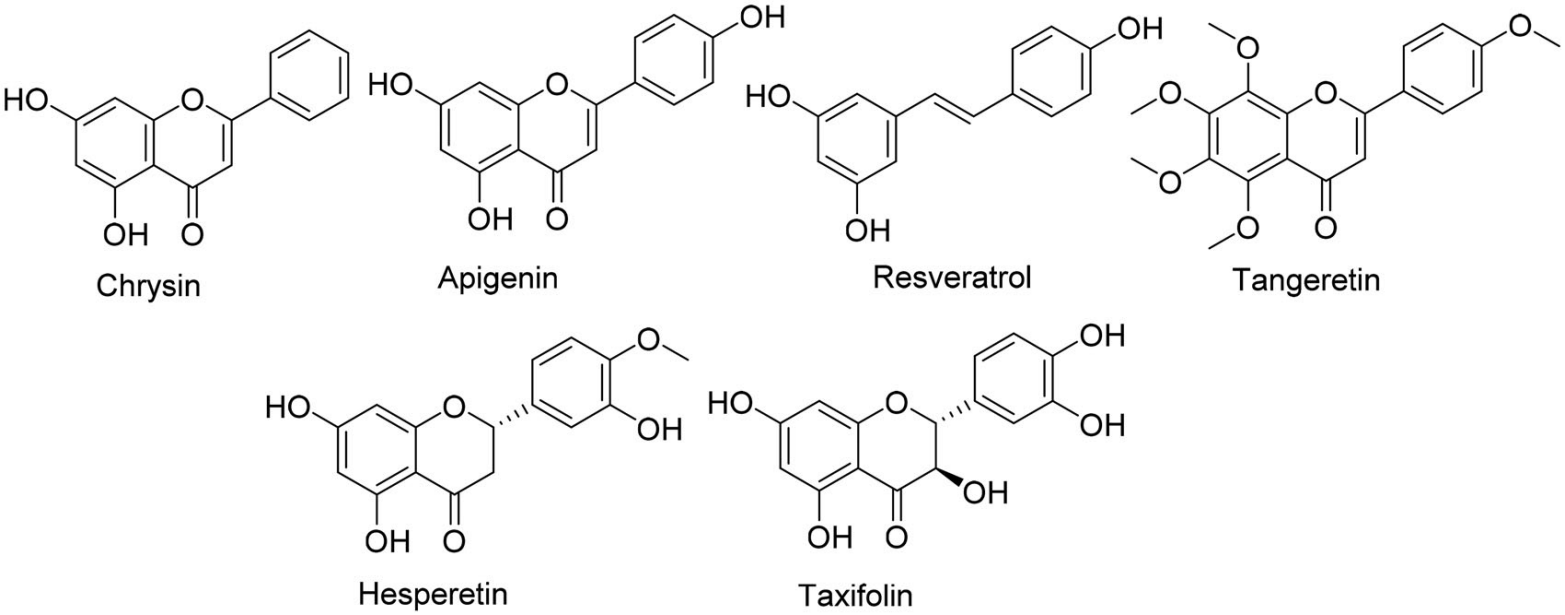
Compounds that showed interaction with HA’s site A.

**Figure 4.**
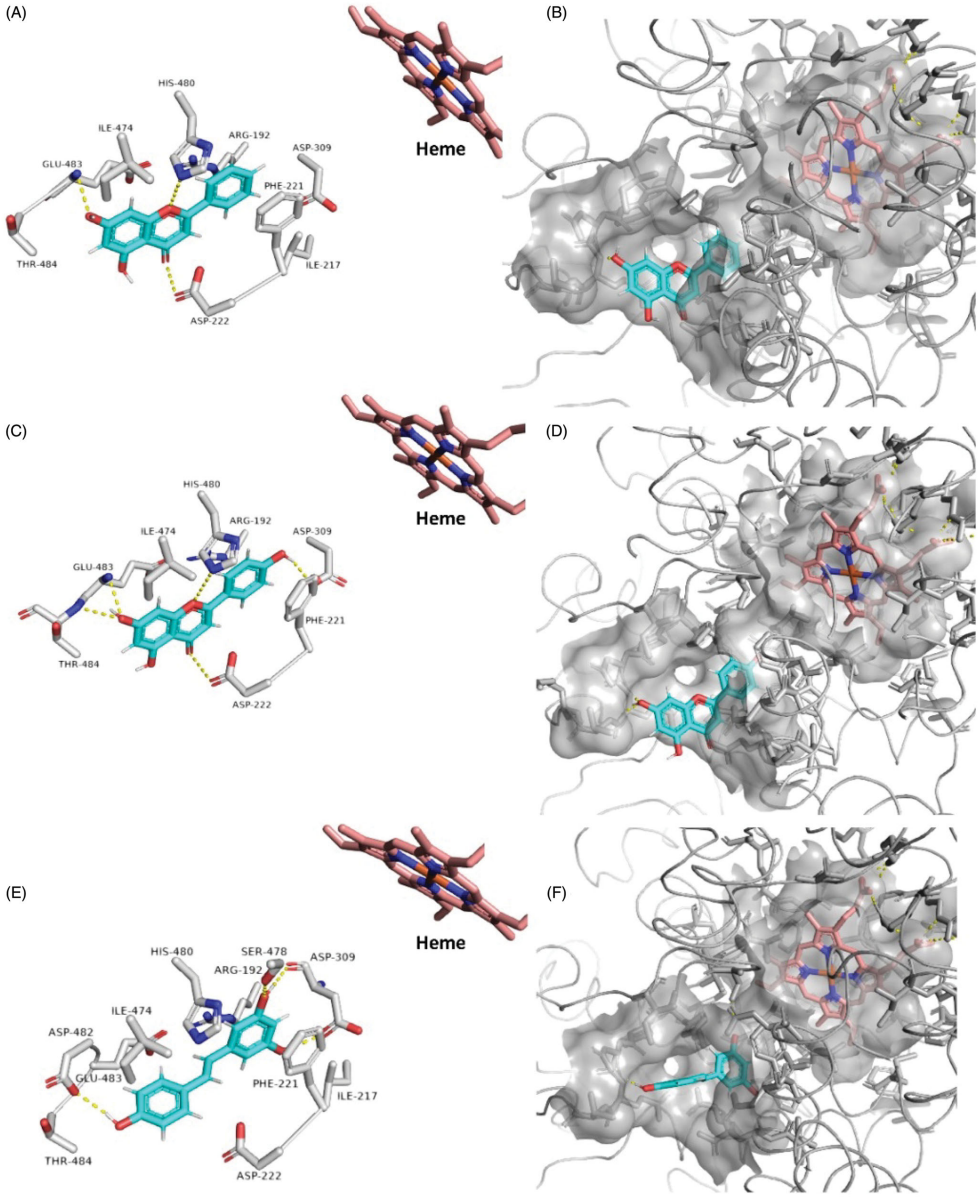
Binding modes of chrysin (A and B), apigenin (C and D), and resveratrol (E and F) inside HA’s substrate access channel after their stabilisation during 100 ns MDS.

We initially docked the whole library (38 out of 52 compounds obeys Lipinski’s rules) against the opening of HA’s substrate access channel’s opening (Site A) using an ensemble docking protocol^39^^,^^40^ to find out the probable active site-directed inhibitors (Table S2). Non-glycosylated flavonoids (9 compounds) alongside resveratrol were found to be the best scoring compounds (< −7 kcal/mol), where the increase in the molecule’s hydroxylation was associated with decreased binding scores. The remaining compounds in the library that got lower scores (> −7 kcal/mol) dissociated from HA’s Site A during the course of 30 ns MDS. The top-scoring compounds were then relaxed by 100 ns-long MDS to get insight into their behaviours inside the binding site and to calculate their binding free energies. The smallest and least hydroxylated flavonoids chrysin, apigenin, and resveratrol (Figure 3) gradually penetrated into site A’s channel towards the haem moiety of the active site. Starting from 56 ns till the end of MDS, their positions remain stable (RMSD ∼0.3 Å for chrysin and apigenin, and ∼1.9 for resveratrol) through multiple interactions (H-bonding, hydrophobic and π-cation interactions). The ring B of both chrysin and apigenin along with the dihydroxylated benzene ring of resveratrol interacted with ARG-192 and PHE-221 via π-cation and hydrophobic interactions, respectively. Besides, the ring C of both flavonoids (chrysin and apigenin) was sandwiched between ASP-222 and HIS-480 through H-bonding, while their ring A was H-bonded to GLU-483 and THR-484. Similarly, the monohydroxylated benzene moiety of the less stable molecule, resveratrol was H-bonded ASP-482 and GLU-483 residues (Figure 4).

**Table 2.** Predicted ADME profiles of the HA inhibitors.

Metabolite	Lipinski^a^	Veber^b^	GIT absorption^c^	BBB^d^	CYP2D6^e^	Bioavailability Score^f^
Resveratrol	Yes	Yes	High	Yes	No	0.55
Chrysin	Yes	Yes	High	Yes	Yes	0.55
Apigenin	Yes	Yes	High	No	Yes	0.55
Pomiferin	Yes	Yes	High	No	No	0.55
Berberine	Yes	Yes	High	Yes	Yes	0.55

^a,b^Predicts if the compound has a drug-like properties (follows the Lipinski’s or Veber’s rules); ^c^predicts the gastrointestinal absorption according to the white of the boiled egg; ^d^predicts the ability of the compound to penetrate the blood–brain barrier (BBB) according to the yolk of the boiled egg; ^e^predicts the cytochrome P450 inhibition; ^f^predicts the bioavailability score, where values >0.5 indicate acceptable bioavailability.

**Figure 5.**
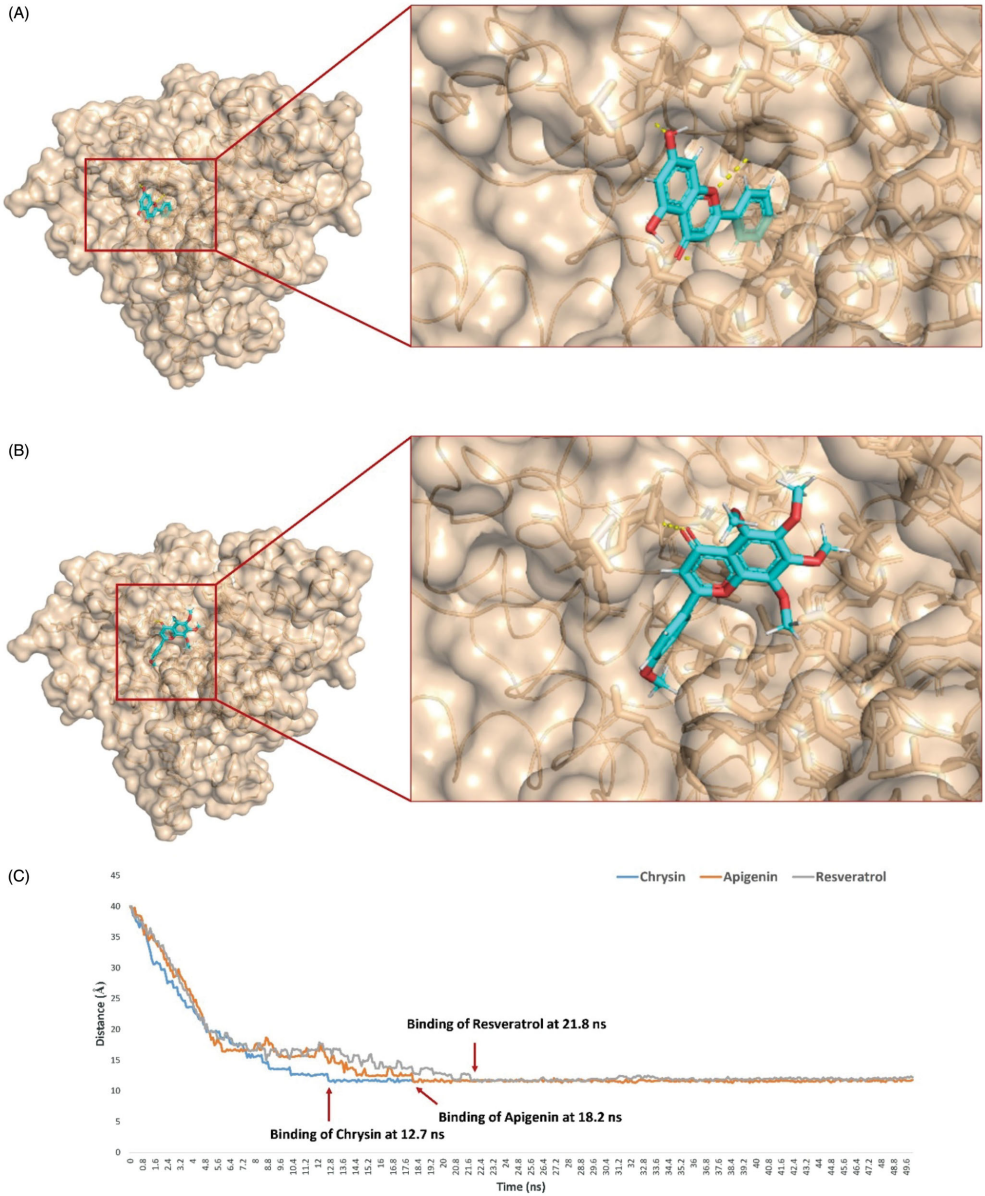
Binding modes of active HA inhibitors (e.g. chrysin; (A) versus inactive ones (e.g. tangeretin; (B) during the 100 ns MDS indicated that the penetration and settling inside the substrate-access channel are essential for the inhibitory activity, where simple flavonoids can penetrate (e.g. chrysin; A), while bulky ones (e.g. tangeretin; B) remained outside and detached from the binding site. (C): Binding process of resveratrol, chrysin, and apigenin, by measuring the distance changes between them and the haem moiety.

On the other hand, the more hydroxylated flavonoids (6 compounds) achieved lower binding free energies (ΔG ∼ −6 kcal/mol) that were also translated in their instability inside the binding site (average RMSD ∼ 10 Å) and significantly lower in-vitro activity (Table 1). We observed during the course of MDS that adding more hydroxyl groups to the flavonoid scaffold hindered them from penetrating the substrate-access channel like the previous derivatives, where they became more involved in multiple transient H-bonds. Moreover, these additional hydroxyl and methoxy groups added extra bulkiness to the molecules making them unable to adopt themselves inside the access channel, and eventually leave the binding site starting from ∼65 ns (e.g. tangeretin, hesperetin, and taxifolin). HIS-475 and HIS-480 were the most frequent residues involved in the H-bonding during the course of MDS, while other the H-bonds were distributed occasionally among other residues outside the access channel (Figures 4 and 5).

To further confirm these findings, we allowed five molecules from each compound to achieve binding events by applying direct forces towards site A during another 50 ns-long simulations. Chrysin, apigenin, and resveratrol were able to achieve bindings after 12.7, 18.8, and 21.8 ns, respectively that were similar to their docking poses. Concerning tangeretin, hesperetin, and taxifolin, they were not able to achieve stable bindings inside site A (Figures 5 and S2). Such structural information may help in designing new HA competitive inhibitors based on the flavonoid scaffold.

### Haem-proximal cavity-directed inhibition (Site B)

Site B occurs in the other side of the HA. It is a wider and shallower (volume = 1083 Å^3^) binding pocket than Site A (Figure 2).It acts as a regulator for HA’s activity by interacting with NADPH cytochrome P450 reductase (CPR) that provide the HA’s haem moiety with the electrons necessary for the reduction process^10^. Hence, targeting this site will lead eventually to a non-competitive-type of HA inhibition.

**Figure 6.**
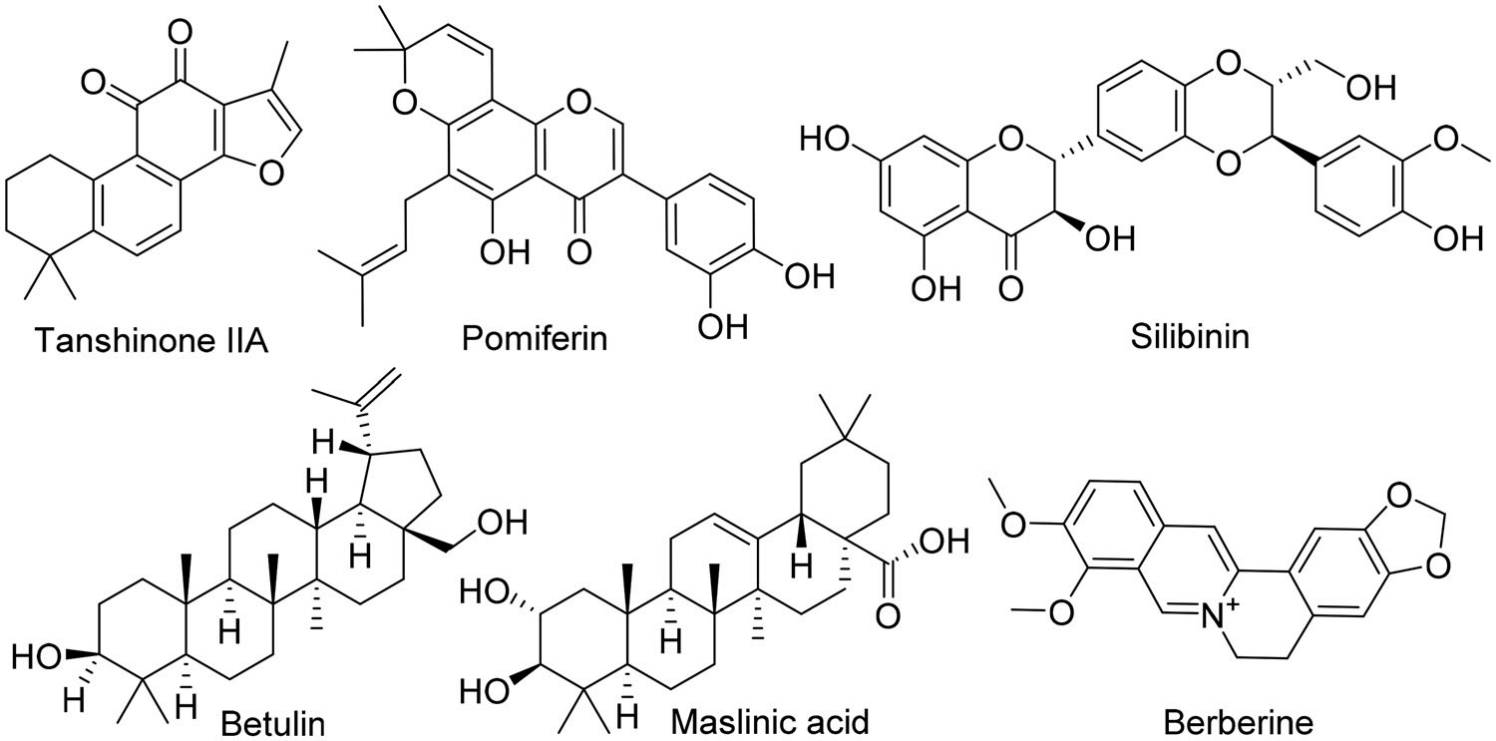
Compounds that showed interactions with HA’s Site B.

**Figure 7.**
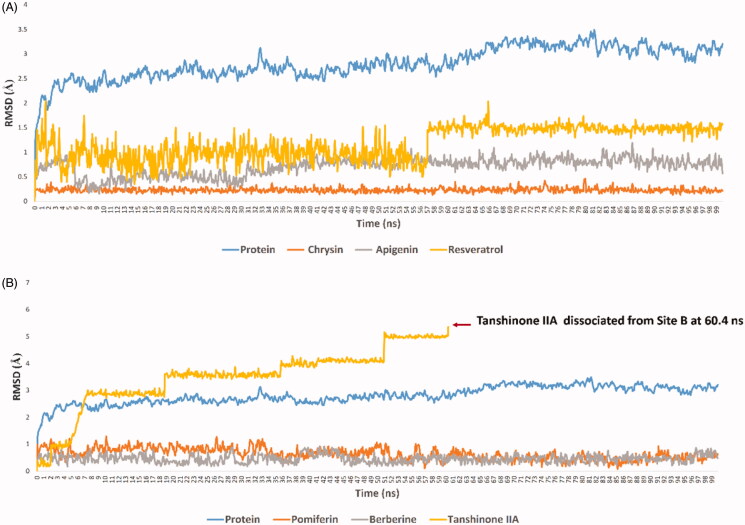
RMSDs of HA together with the best scoring inhibitors inside its Site A (A) and Site B (B).

**Figure 8.**
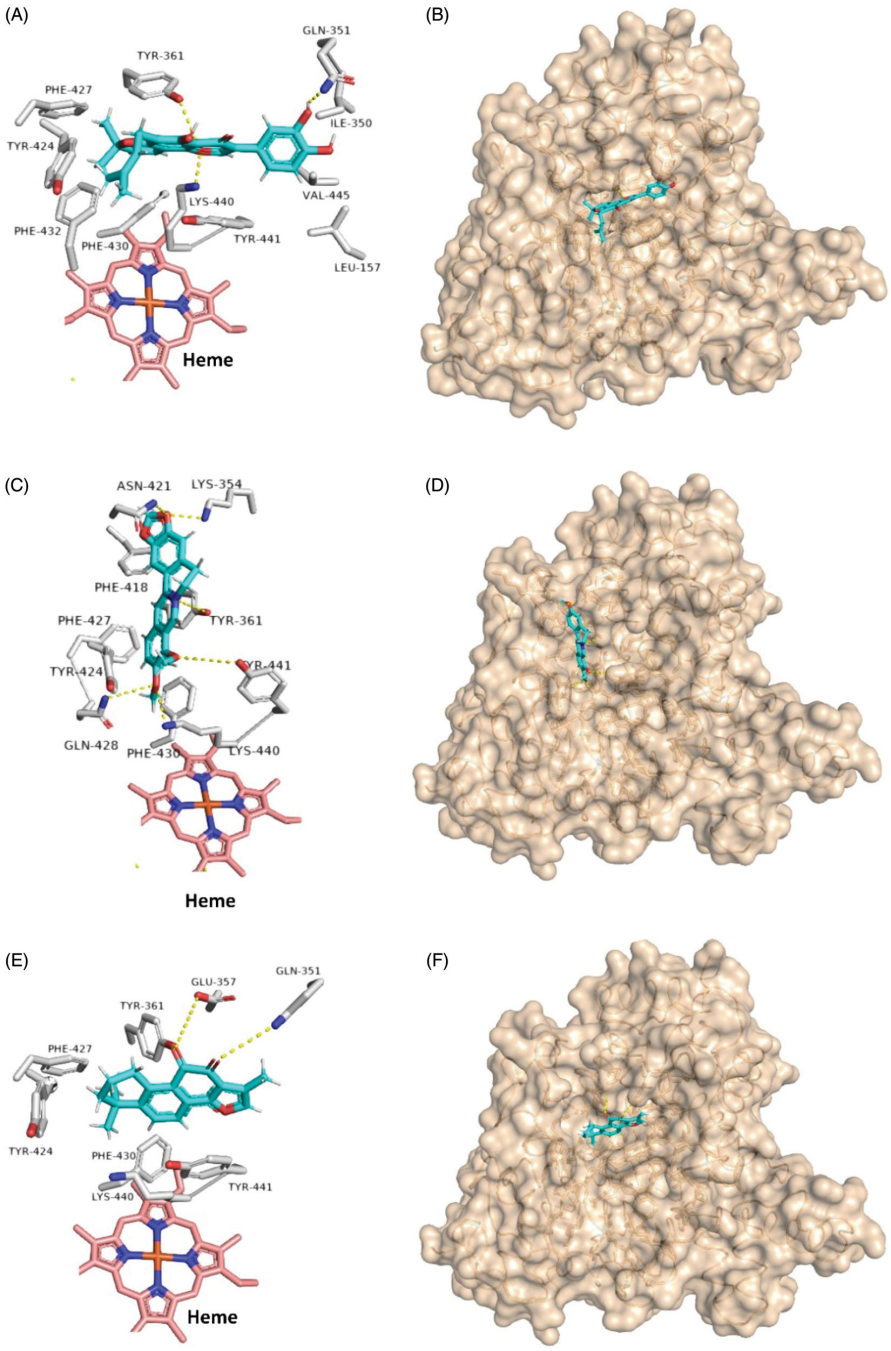
Binding modes of pomiferin (A and B), berberine (C and D), and tanshinone IIA (E and F) inside HA’s haem-proximal cavity after their stabilisation during 100 ns MDS.

We applied the same protocol for the haem-proximal cavity (site B), where all compounds in our in-house library were docked against this allosteric binding cavity (Table S2). Only tanshinone IIA, pomiferin, silibinin, betulin, maslinic acid, and berberine got binding scores < −7.5 kcal/mol (Figure 6). Lower scoring compounds (46 compounds) showed rapid dissociation from the HA’s site B after the first 5–10 ns of MDS. Moreover, silibinin, botulin and maslinic acid have completely left the binding site (i.e. Site-B) after ∼ 20 ns. Further, 100 ns-long MDS were applied for these three top-scoring compounds (tanshinone IIA, pomiferin, and berberine) to reveal their interactions inside site B and to calculate their binding free energies (Figure 7). Pomiferin got the highest binding free energy (ΔG = −10.3 kcal/ mol) which reflects the highest stability inside the binding cavity. This compound remained settled during the course of MDS (RMSD ∼ 0.6 Å) through three strong H-bond interactions (<2.5 Å), one of them was between GLN-351 and one of the two hydroxyl groups of ring B, and the remaining ones were between both TYR-361, LYS-440 and ether group of ring C. The remaining part of the compound (ring A and its hydrophobic extension) was embedded in a hydrophobic pocket consisted of TYR-361, TYR-424, PHE-427, PHE-430, and PHE-432 (Figures 7 and 8). Berberine achieved the second-highest binding free energy (ΔG = −9.3 kcal/mol) and stability (RMSD ∼ 0.8 Å). It got an orientation slightly different from that of pomiferin inside the binding site, where the four etheric oxygen on both sides of the molecule scaffold were involved in H-bonding with LYS-354, ASN-421, TYR-424, and TYR-441, respectively. Additionally, TYR-361 was H-bonded to the positively charged nitrogen. Furthermore, the aromatic body of the whole molecule showed multiple hydrophobic interactions with TYR-361, PHE-418, PHE-427, and PHE-430 (Figures 7 and 8). On the other hand, tanshinone IIA got significantly lower binding free energy (ΔG = −6.2 kcal/mol), and achieved less stable binding (RMSD ∼ −5.9 Å till 60.4 ns). At 51 ns, it started to leave the binding site and finally reached complete dissociation at 60.4 ns. This obvious instability could be explained by the absence of strong binding interactions between the molecule and the amino acid residues inside the binding site. Even the rare H-bondings that have recorded during the MDS were very weak (∼ 4.7 Å) (Figures 7 and 8).

**Figure 9.**
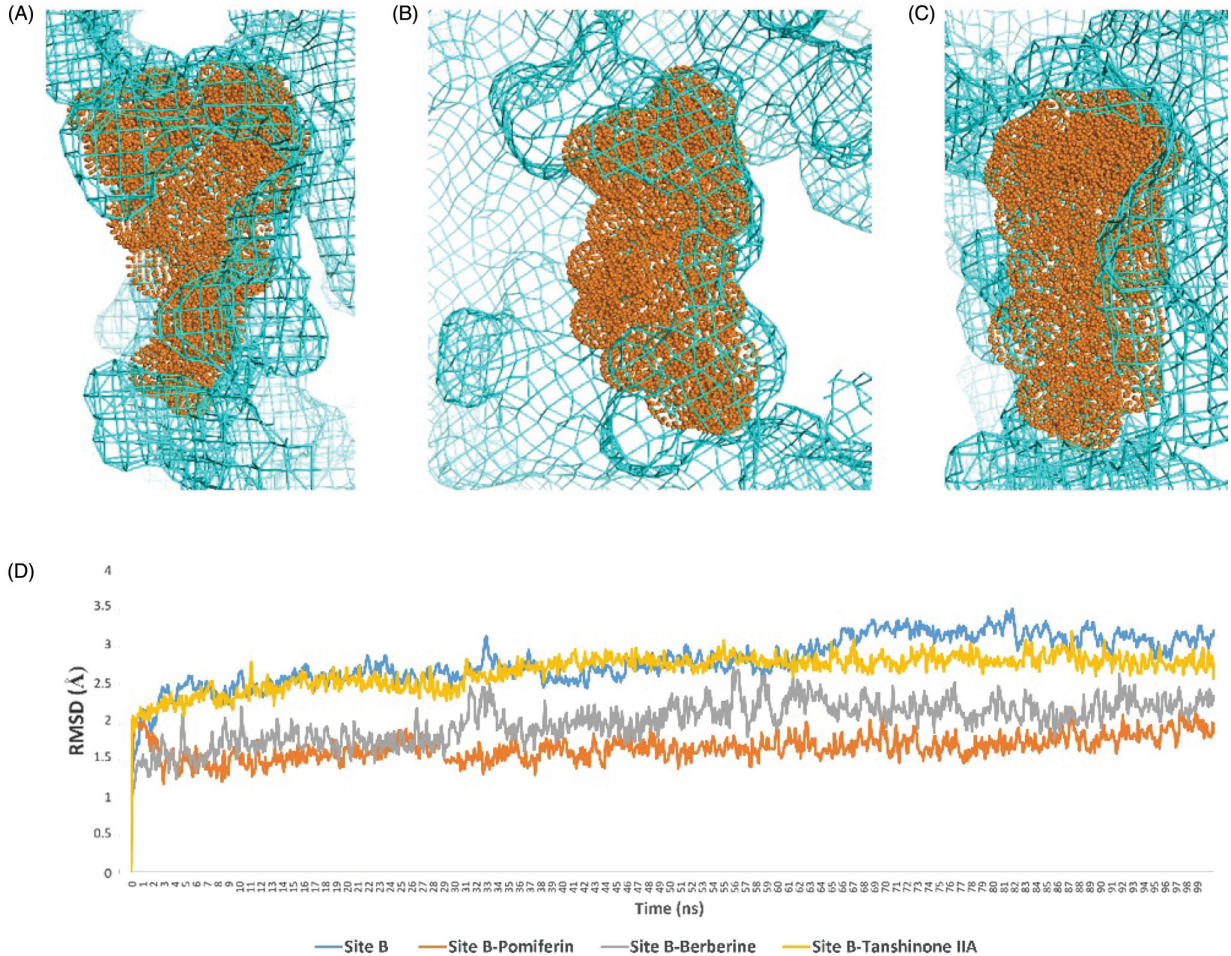
Differences in the degree of ligand-binding site shape complementarity between pomiferin, berberine, and tanshinone IIA (A, B, and C, respectively), and RMSDs of Site B in the presence of the same ligands (D).

Moreover, we assessed the bindings of pomiferin, berberine, and tanshinone IIA with Site B by estimating their shape complementarity scores (*S*_c_ ; Figure 9(A–C)). Pomiferin showed the highest degree of shape complementarity with the binding site (i.e. Site B) followed by berberine and tashinone IIA (*S*_c_ = 0.75, 0.69, and 0.48, respectively). Consequently, the side chains of Site-B’s aminoacids achieved the highest stability during the course of MDS in case the binding with pomiferin followed by berberine, while with tanshinone IIA, its average RMSD was almost identical to that of the free unbounded form (Figure 9(D)).

### Experimental validation

To validate our preliminary computational experiments, top-scoring compounds were tested for their HA inhibitory activity and their antiproliferative effect against both the ER + MCF-7 and the ER- MDA-MB-231. Additionally, we performed enzyme kinetic analyses to calculate the inhibition constant (*K*_i_) and the type of inhibition of each HA inhibitor^41^.

As shown in Table 1, chrysin, apigenin, and resveratrol that got the highest binding free energies values (*ΔG* ∼ −9.7 kcal/mol) were also the most potent HA inhibitors and were more active against MCF-7 than MDA-MB-231. Also, they were found to inhibit HA via a competitive mechanism, and hence such results were in good accordance with the site A-directed inhibition as the computational investigation suggested. Moreover, tangeretin that was unstable inside site-A was inactive against HA. However, it showed some activity against both MCF-7 and MDA-MB-231, indicating that it might deal with cancer cells through a different mode of action.

Regarding compounds that were suggested to target site-B, both pomiferin and berberine were the best HA inhibitors, while the less stable one, tanshinone IIA was far less active. MCF-7 cell line was also more sensitive to the former compounds than MDA-MB-231. Surprisingly, tanshinone IIA was the most active compound against both cell lines (IC_50_ ∼ 2.1 µM), and such observation suggested a different mechanism of action for this compound. A previous study has reported that tanshinone IIA can inhibit both breast cancer cell lines *via* targeting ADP-ribosyltransferase like protein 1 (ADPRTL1)^42^. Betulin that detached from site 2 after 20 ns showed correspondingly very weak inhibitory activity against HA and both breast cancer cell lines. To further test the efficacy of our virtual screening protocol, we have tried two of the lowest scoring compounds; abscisic acid and rutin (*ΔG* > −3 kcal/mol), and both of them were inactive either against HA or breast cancer cell lines.

### In silico drug-like properties and ADME prediction

Compounds that showed HA inhibitory activities (Table 2) were further assessed for their drug-like properties. Additionally, their Absorption, Distribution, Metabolism, Excretion (ADME) profiles were calculated. In-silico-based estimation of the physicochemical properties (e.g. molecular weight and log *P*) of certain bioactive compounds could suggest its probable pharmacokinetics. Lipinski’s rule of five contemplates a small bioactive molecule as a drug candidate if it possesses these physicochemical parameters i.e. log *P* ≤ 5, molecular weight ≤500, hydrogen bond donor [HBD] ≤ 5, and hydrogen bond acceptor [HBA] ≤ 10. Hence, around 90% of orally active drugs that have passed phase 2 clinical trials obey such rules^43^. Drug’s cellular permeability and its distribution and excretion are linked to their molecular flexibility and topological polar surface area (tPSA). Consequently, bioactive molecules with tPSA of 140 Å or less and rotatable bonds of ten or fewer (i.e. Veber’s oral bioavailability) can also be considered as potential drug candidates^43^.

The ADME profiles of the active metabolites resveratrol, chrysin, apigenin, pomiferin, and berberine were calculated by the online software SwissADME. As illustrated in Table 2, all of them were found to obey both Lipinski’s and Veber’s rules of drug-likeness. Moreover, they were predicted to exhibit good bioavailability. Hence, these metabolites could be considered as promising candidates for further in vivo evaluation as potential drug leads or even dietary supplements for the management of ER + breast cancer.

## Discussion

BC remains a significant worldwide health issue, particularly for women. Besides the standard treatment protocols that rely on chemotherapeutic agents, ER + BC can be managed by other therapeutics that modulate oestrogen. Both SERMs and ArIs have introduced as good options in this regard; however, their prolonged use was associated with acquired resistance^44^. Being the more effective class with fewer side effects, ArIs have gained much interest in the last two decades, when several generations of ArIs have been developed. All of these inhibitors, either steroidal or non-steroidal, were designed to target the enzyme active site. Recently, experimental and computational investigations of this enzyme have revealed other potential targets on this protein that can be considered for the development of the next generation inhibitors that can handle the issue of resistance^10^^,^^11^.

Plant-based natural products are still a crucial and diverse source of potential therapeutics and nutraceuticals that have a good safety profile. Many plant-derived compounds have been reported as very good ArIs, particularly, flavonoids and other phenolic compounds^12^^,^^15^. However, their exact molecular interaction remained to be explored.

Herein, we applied an *in silico*-based protocol to identify the most promising HA inhibitors depending on their site of interaction. In our initial virtual screening, we focussed on compounds that can target the enzyme’s unusual binding sites (i.e. substrate access channel opening and haem-proximal cavity). Subsequently, a series of MDS experiments were performed to refine our preliminary docking step and to explore the mode of interaction of the best candidates. Non-glycosylated flavonoids along with resveratrol were found to be the best hits for site-A (i.e. the opening of substrate access channel) that mediates the competitive-type of HA inhibition, and upon MDS, resveratrol, chrysin, and apigenin were found to settle inside the channel achieving the highest binding free energies.

Despite these three compounds have been previously reported as HA competitive inhibitors, their exact mode of interaction with HA was illusive^44^^,^^45^. Consequently, future structural-based drug screening studies can utilise this scaffold of these compounds and the substrate access channel to discover further potential inhibitors.

On the other hand, tanshinone IIA, pomiferin and berberine were found to be the best ligands for site B (i.e. haem-proximal cavity) that mediates the non-competitive-type of HA inhibition. However, only pomiferin and berberine were able to achieve stable binding inside this binding cavity with high bending free energies during the course of 100 ns MDS. The subsequent experimental validation came to support the *in silico* investigation. All of the best-scoring hits showed micromolar inhibition of HA, where site-A-targeting hits were more potent than those targeting site-B. Moreover, site A-directed inhibition led to competitive inhibition, while site-B-directed inhibition led to a non-competitive one. The ER + BC cell line MCF-7 was also more sensitive to these inhibitors than the ER- one MDA-MB-231.

Despite being less potent against HA, site B-directed non-competitive inhibitors offered some advantages over the site A-directed competitive one: (i) they were able to bypass the developed resistance against active-site directed inhibition in which the enzyme mutates the binding domain to be inaccessible for the inhibitor, (ii) they will not abolish oestrogen production completely and thus the onset of resistance can be delayed, (iii) such allosteric inhibitors offer some selectivity over the active site ones, and hence, they might be with much lower side effects^46^.

Both pomiferin and berberine have been shown anti-breast cancer activity^47^, and pomiferin was reported to inhibit HA^48^. However, our current investigation illustrates their unusual interactions with HA’s allosteric site laying the foundation for further development of more potent and specific derivatives.

Besides being good HA inhibitors, they also showed good bioavailability and drug-like properties according to both Lipinski’s and Veber’s rules of drug-likeness. Consequently, these plant-derived compounds along with the structural information presented in this study offer a good starting point for further development of more effective inhibitors via structural modification or the subsequent *in vivo* testing to be the next generation of ArIs.

In conclusion, we introduced an effective workflow for the rapid identification of HA inhibitors describing their possible modes of action. Hence, using this protocol with more extensive databases can help in the discovery of different HA inhibitors, particularly the non-competitive allosteric ones that can be promising alternatives or supportive to the current ArIs.

## Supplementary Material

Supplemental Material
