# Supplementary material for: Targeting allosteric sites of human aromatase: a comprehensive in-silico and in-vitro workflow to find potential plant-based anti-breast cancer therapeutics
Source: J Enzyme Inhib Med Chem. 2021 Jun 17;36(1):1333–44. doi: 10.1080/14756366.2021.1937145 (PMC8759730; doi:10.1080/14756366.2021.1937145)
Supplement: Supplemental Material [file IENZ_A_1937145_SM2485.pdf]

Supplementary Material

Targeting Allosteric Sites of Human Aromatase: A *comprehensive in-silico* and *in-vitro* Workflow to Find Potential Plant-based Anti-breast Cancer Therapeutics.

Hani A. Alhadrami[1,2,3†], Ahmed M. Sayed[4,†], Sami A. Melebari[3], Asem A. Khogeer[5], Wesam H. Abdulaal[6], Mohamed B. Al-Fageeh[7], Mohammad Algahtani[8] and Mostafa E. Rateb[9,*]

[1] Department of Medical Laboratory Technology, Faculty of Applied Medical Sciences, King Abdulaziz University, P. O. Box 80402 Jeddah 21589, Saudi Arabia.

[2] Molecular Diagnostic Lab, King Abdulaziz University Hospital, King Abdulaziz University, P. O. Box 80402 Jeddah 21589, Saudi Arabia

[3] Molecular Diagnostic Unit, The Regional Laboratory in Makkah, Ministry of Health, Kingdom of Saudi Arabia

[4] Department of Pharmacognosy, Faculty of Pharmacy, Nahda University, 62513 Beni Suef, Egypt;

[5] Plan and Research Department, General Directorate of Health Affairs, Makkah region, Ministry of Health

[6] Department of Biochemistry, Faculty of Science, King Abdulaziz University, Jeddah, Saudi Arabia

[7] General Directorate for Funds and Grants (GDFG), King Abdulaziz City for Science and Technology, P. O. Box 6086, Riyadh 11442, Saudi Arabia.

[8] Department of Laboratory and Blood Bank Security Forces Hospital Program , Mecca, Saudi Arabia.

[9] School of Computing, Engineering & Physical Sciences, University of theWest of Scotland, Paisley PA1 2BE, UK.

† Authors are equally contributed as first author to this work

* Correspondence to Mostafa Rateb: email Mostafa.Rateb@uws.ac.uk, Phone: +44141 848 3072.

Table S1. Coordinates and amino acid residues of Sites A and B.

	Grid Box (Å)	Amino acid Residues
**Site A**	center_x = 86.3<br>center_y = 51.1<br>center_z = 55.6	ARG-192, VAL-214, VAL-215, ILE-217, GLN-218, GLY-219, PHE-221, ASP-222, PRO-308, ASH-309, SER-312, VAL-313, ILE-474, HIS-480, PRO-481, ASP-482, GLU-483, THR-484
**Site B**	center_x = 74.3<br>center_y = 39.1<br>center_z = 35.1	LEU-157, ILE-350, GLN-351, LYS-354, GLU-357, TYR-361, PHE-418, ASN-421, VAL-422, TYR-424, PHE-427, GLN-428, PRO-429, PHE-430, GLY-431, PHE-432, Lys440, TYR-441, MET-444, LYS-448

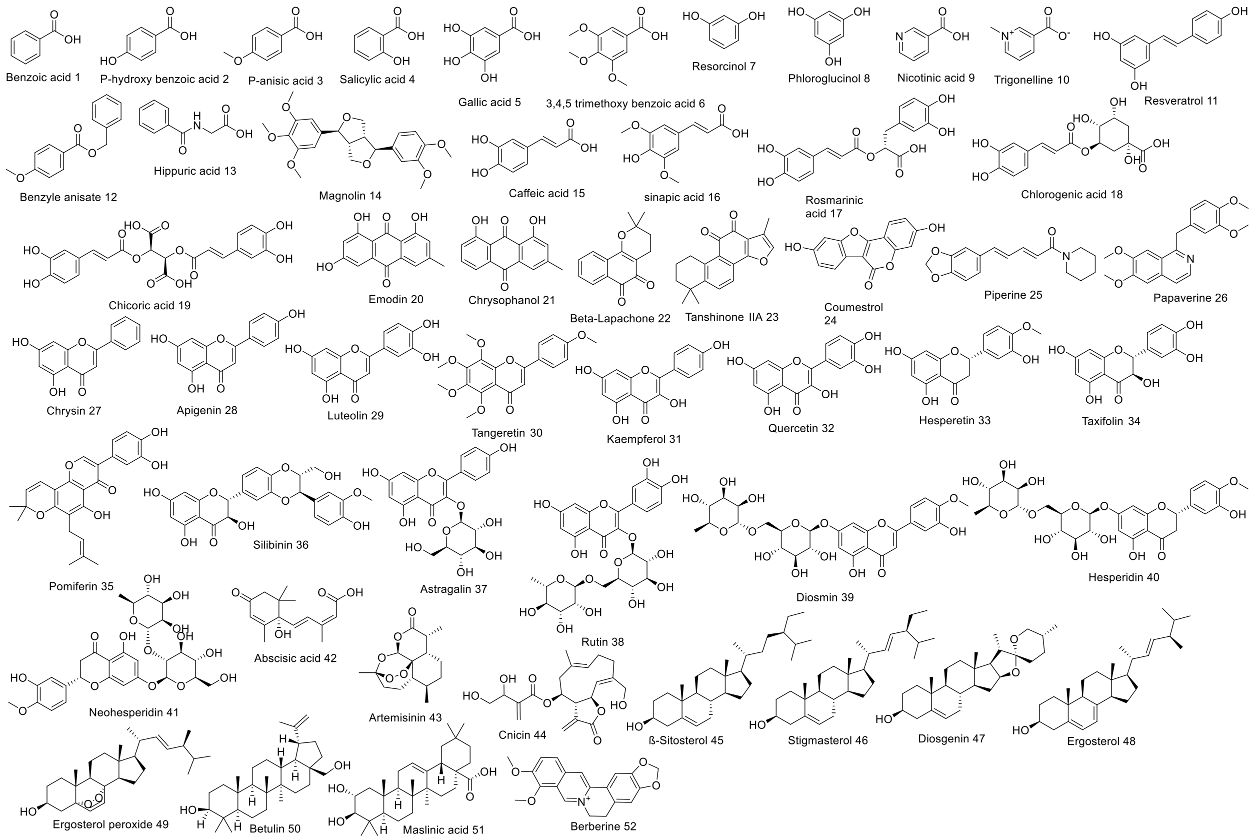

Figure S1. Compounds 1-52 of the plant-derived library used in this study.

Table S2. Docking scores and binding free energies of the library's compounds along with their calculated drug-likeness according to Lipinski's rules.

**No.**	**Compound**	**Average ΔG^{Vina}**		**ΔG^{FEP}**		**Lipinski**
		Site A	Site B	Site A	Site B	
1	Benzoic acid	-3.7	-3.2	-1.2	-0.8	Yes
2	*p*-Hydroxy benzoic acid	-3.8	-2.9	-1.7	-1.1	Yes
3	*p*-Anisic acid	-3.6	-3.3	-1.5	-0.9	Yes
4	Salicylic acid	-3.7	-2.8	-1.4	-0.9	Yes
5	Gallic acid 5	-3.9	-3.0	-2.2	-1.8	Yes
6	3,4,5 trimethoxy benzoic acid 6	-3.8	-3.2	-2.9	-1.5	Yes
7	Resorcinol	-3.2	-2.9	-1.5	-1.1	Yes
8	Phloroglucinol	-2.9	-2.8	-1.8	-1.3	Yes
9	Nicotinic acid	-3.3	-3.0	-1.9	-1.5	Yes
10	Trigonelline	-3.5	-3.2	-1.5	-1.7	Yes
11	**Resveratrol**	**-10.4**	-5.6	**-8.2**	-3.9	Yes
12	Benzyle anisate	-6.5	-5.9	-4.9	-4.2	Yes
13	Hippuric acid	-6.1	-6.1	-4.8	-3.8	Yes
14	Magnolin	-6.5	-6.5	-4.8	-4.3	Yes
15	Caffeic acid	-6.3	-5.8	-4.2	-3.7	Yes
16	Sinapic acid	-6.9	-6.1	-4.5	-3.6	Yes
17	Rosmarinic acid	-6.5	-7.0	-4.9	-4.9	Yes
18	Chlorogenic acid	-6.3	-7.2	-4.2	-4.9	No
19	Chicoric acid	-6.2	7.5	-4.5	-5.0	No
20	Emodin	-6.5	-6.2	-4.1	-4.1	Yes
21	Chrysophanol	-6.8	-6.3	-4.9	-4.2	Yes
22	Beta-Lapachone	-6.7	-7.1	-4.8	-4.9	Yes
23	**Tanshinone IIA**	-6.9	**-7.8**	-4.7	**-6.2**	Yes
24	Coumestrol	-6.8	-7.2	-4.2	-5.0	Yes
25	Piperine	-5.8	-6.5	-4.1	-4.3	Yes
26	Papaverine	-6.5	-6.9	-4.2	-4.5	Yes
27	**Chrysin**	**-10.1**	-6.8	**-8.1**	-4.4	Yes
28	**Apigenin**	**-10.0**	-6.8	**-8.2**	-4.5	Yes
29	Luteolin	-8.1	-6.9	-6.5	-4.7	Yes
30	Tangeretin	-7.9	-6.9	-5.9	-4.4	Yes
31	Kaempferol	-8.6	-6.8	-6.2	-4.5	Yes
32	Quercetin	-8.2	-6.8	-6.3	-4.4	Yes
33	Hesperetin	-7.9	-6.9	-6.0	-4.3	Yes
34	Taxifolin	-7.8	-6,8	-6.1	-4.6	Yes
35	**Pomiferin**	-6.5	**-13.9**	-4.4	**-10.3**	Yes
36	Silibinin	-6.9	-7.5	-5.0	-5.2	Yes

37	Astragalin	-6.1	-7.2	-4.1	-4.7	No
38	Rutin	-5.7	-7.0	-3.2	-4.8	No
39	Diosmin	-5.5	-7.1	-3.3	-4.9	No
40	Hesperidin	-5.8	-7.0	-3.6	-4.8	No
41	Neohesperidin	-5.1	-7.0	-3.2	-4.9	No
42	Abscisic acid	-4.3	-4.9	-3.4	-3.3	Yes
43	Artemisinin	-4.7	-5.2	-2.9	-3.8	Yes
44	Cnicin	-6.8	-7.3	-4.9	-5.0	Yes
45	ß-Sitosterol	-6.9	-6.2	-4.6	-4.3	No
46	Stigmasterol	-6.8	-6.1	-4.6	-4.1	No
47	Diosgenin	-6.8	-6.5	-4.4	-4.4	No
48	Ergosterol	-6.9	-6.4	-4.9	-4.2	No
49	Ergosterol peroxide	-6.7	-6.6	-4.9	-4.5	No
50	Betulin	-6.7	-6.7	-4.6	-4.6	No
51	Maslinic acid	-6.8	-6.8	-4.3	04.9	No
52	**Berberine**	-5.8	**-13.1**	-3.9	**-9.3**	Yes

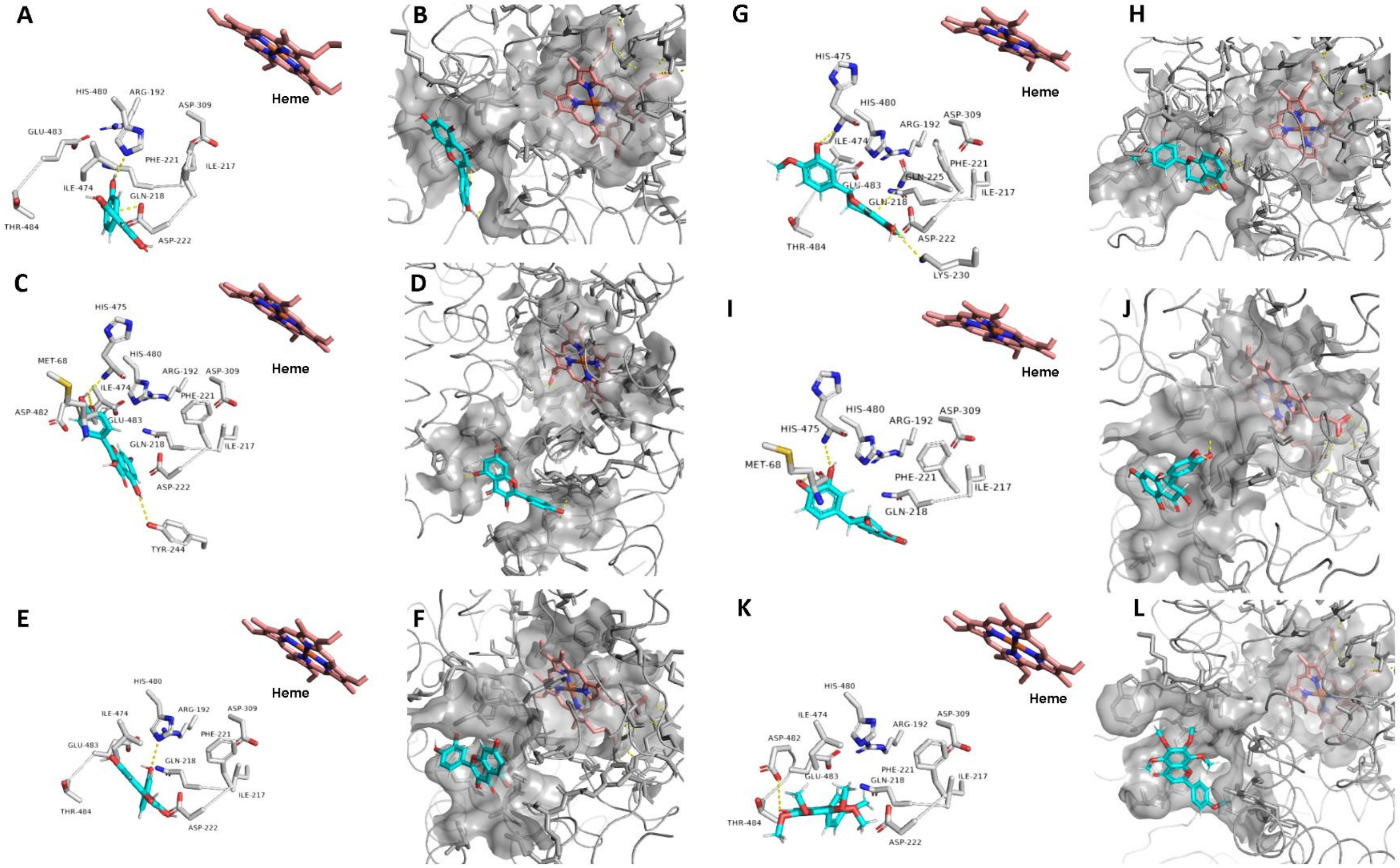

Figure S2. Binding modes of inactive HA inhibitors during the 100 ns MDS indicated that the penetration and settling inside the substrate-access channel are essential for the inhibitory activity. Hydroxylated flavonoids (compounds 29-34) remained outside and detached from the binding site.
